# Psychological resilience on the frontlines: Operational Resilience Training for Ukrainian combat medics

**DOI:** 10.1080/08995605.2026.2698195

**Published:** 2026-07-02

**Authors:** Andreas Espetvedt Nordstrand, Haakon Gabrielsen Engen, Line Rønning, Christer Lunde Gjerstad, Amy B. Adler

**Affiliations:** a Section for Military Mental Health, Joint Medical Forces, Norwegian Armed Forces, Oslo, Norway; b Department of Psychology, Norwegian University of Science and Technology (NTNU), Trondheim, Norway; c Department of Psychological Sciences, Northern Arizona University (NAU), Flagstaff, Arizona, USA; d Norwegian Defence University College, Royal Norwegian Air Force Academy, Trondheim, Norway; e Department of Research, Division of Mental Health and Addiction, Oslo University Hospital, Oslo, Norway; f Center for Military Psychiatry and Neuroscience, Walter Reed Army Institute of Research (WRAIR), Silver Spring, Maryland, USA

**Keywords:** Operational Resilience Training, combat medics, psychological resilience, Ukraine, large-scale combat operations

## Abstract

On the battlefield, combat medics need to both support service members’ physical health, and their psychological resilience and mental health. Despite the importance of their role, combat medics are typically not trained in resilience and mental health, and for units mobilized rapidly in response to a country-wide threat, the need to address this gap is particularly critical. Consistent with this challenge, Operational Resilience Training (ORT) was developed by a combined Norwegian-US initiative to provide Ukrainian combat medics with psychological resilience skills necessary for frontline service. ORT was evaluated using surveys before and after the two-day training (*N* = 145); a sub-sample was also surveyed again one month later (*n* = 39). Findings indicated more than 90% of participants found the training to be useful and relevant, and that participants intended to use the skills they learned when returning to combat. Moreover, there was a significant and temporally stable increase in trainees’ confidence in psychological self-care and peer support abilities. The results highlight the importance of integrating resilience training into military medical curricula, particularly for medics operating at the frontlines. These insights may inform the development of resilience programs across NATO and allied forces facing similar operational demands.

**What is the public significance of this article?—**Combat medics in large-scale combat operations may need practical tools to support both physical survival and psychological functioning at the frontlines. This study suggests that Operational Resilience Training was viewed as useful and relevant by Ukrainian combat medics and was associated with increased confidence in using mental skills for self-care and peer support. These findings may help military organizations develop scalable resilience training for medics and other frontline personnel, while underscoring the need for future studies using performance-based outcomes.

For service members, deployment to a combat zone constitutes one of the toughest occupational challenges. The relevant stressors range from daily hardships of sustained frontline deployment to direct exposure to potentially traumatic combat events (e.g., Anyan et al., 2024; Brusher, 2011; Gibbons et al., 2012). In Ukraine, the distinction between deployment and combat stressors may be especially blurred. Drone-enabled surveillance and attacks, artillery, and strikes on civilian and energy infrastructure can extend threat beyond conventional firefights or fixed frontlines, and make resupply and rotation of forces both perilous and logistically difficult (Bongartz et al., 2026; Hukovskyy et al., 2024). Moreover, numerous studies have documented the negative impact of combat stressors on the mental and physical health of service members (e.g., Adler et al., 2011; Bryan et al., 2015; Hoge et al., 2014; Levy & Sidel, 2009; Nordstrand et al., 2019; Thomas et al., 2017), and how a high burden of deployment-related stressors like lack of privacy, austere living conditions and separation from family can potentiate the deleterious effects of combat experiences (e.g., Huffman et al., 2025). Yet, within the context of both combat and deployment-related stressors, service members are expected to perform their mission and protect fellow unit members.

Military preparation is a major factor influencing the ability of service members to withstand combat and deployment stressors. Tactical training, foundational military skills, mental preparedness, and unit-level resources have all been identified as protective factors for coping with the stress of military operations (e.g., Adler & Castro, 2013; DiGangi et al., 2013; Kalisch et al., 2017; Kok et al., 2020; Xue et al., 2015). However, if countries must rapidly mobilize their civilian population in response to a substantial threat, there may not be much time for military training and mental preparation. As a result, both the tactical skills and combat mind-set of service members could be underdeveloped. Accordingly, rapidly mobilized service members risk cognitive overload in the high-stress, high-demand environments typical of military engagements (Stein et al., 2021; Tornero-Aguilera et al., 2024) and large-scale combat operations (LSCO), potentially increasing susceptibility to peri-traumatic over-activation and impaired performance under fire (e.g., Jensen et al., 2020; Patton & Rubinstein, 2023). Moreover, inadequate training and mental preparedness can contribute to a greater likelihood of stress-induced mental health challenges long term (Huffman et al., 2025; Lensvelt-Mulders et al., 2008).

With Russia’s full-scale invasion of Ukraine in February 2022, Ukraine was faced with the need to rapidly mobilize its population to counter this existential threat (e.g., Nordstrand, Barger, et al., 2024). Although some Ukrainians had prior military experience due to Russia’s annexation of Crimea in 2014 and its support for separatist forces in Eastern Ukraine, the response to the 2022 invasion required accelerated and abbreviated training (International Institute for Strategic Studies, 2023) in order to meet the demands of LSCO. Since then, fighting has remained intense, frontline deployment has been essentially continuous, and casualty rates have been notably high (Council on Foreign Relations, 2026).

Collectively, these realities fuel the need for resilience tools that can help service members manage the cognitive and emotional toll associated with combat and deployment stressors. Such tools can be provided as real-time prevention measures. Moreover, rather than add to the training burden during the accelerated pre-deployment phase for new recruits and conscripts, resilience tools can be provided to combat medics. Combat medics are embedded into units at the frontline and can serve as force multipliers by not only treating service members who are injured but also disseminating stress control techniques. In Ukraine, medics are particularly vital because there are few other medical assets on the front lines (Hukovskyy et al., 2024). Their role is also important in other Western countries where medics may serve as the first line of defense in helping units manage psychological stress (Hoyt & Hein, 2021; Marsh & Hampton, 2022). Despite the central role of combat medics in addressing psychological stress, they typically do not receive training focused on mental health, psychological resilience and stress management (Mesias et al., 2024).

Recognizing the need for better preparation of combat medics, the Ukrainian Armed Forces requested that their European partners provide comprehensive training in mental health and resilience as part of the combat medic curriculum being offered by European and US counterparts. In response, the Norwegian Armed Forces, in collaboration with the Walter Reed Army Institute of Research (WRAIR), developed Operational Resilience Training (ORT), a program specifically tailored for Ukrainian combat medics. ORT was based on WRAIR’s extensive research on resilience training (Adler & Gutierrez, 2022; Adler et al., 2015, 2020; Cacioppo et al., 2015; Crouch et al., 2024; Mesias et al., 2024; Nordstrand et al., 2026; Nugent et al., 2024), incorporating structured modules that focus on both individual and unit-level stress management techniques. The techniques were designed for service members to apply both when helping themselves and when helping others. Just as with the combat lifesaver framework (Schauer et al., 2023), ORT enables combat medics to serve as a vector for teaching skills about self-care and buddy care that is analogous to the central role they have in disseminating knowledge on medical first aid.

The present study evaluated Ukrainian combat medics’ ratings of ORT and changes in their confidence from pre- to post-training. Specifically, the goal of this study was to assess perceptions of the ORT program’s acceptability, the degree to which the training was associated with increased confidence in targeted domains, and, using preliminary data, the extent to which confidence was maintained over the course of a month. Findings from this evaluation can inform resilience training efforts across allied forces.

## Method

This study was approved by the ethical review board of the Norwegian Armed Forces Joint Medical Services (NAFJMS). Potential participants were briefed on the purpose of the study and asked for their written informed consent, which was provided by 98.2% (273/278). Procedures, including data collection, storage, and distribution, were conducted in accordance with NAFJMS data governance regulations.

### Study design and procedure

Three surveys were administered during the five-week combat medic course from spring 2023 through spring 2024. ORT training was provided in the first week of the course. The ORT program consisted of two days of classroom-based instruction with practical exercises targeting specific ORT skills. The following week, the combat medic course included a one-day scenario-based field exercise designed to reinforce ORT skills in a simulated operational environment (Nordstrand et al., 2026). The rest of the combat medic course covered advanced principles of military medical operations, including Tactical Combat Casualty Care, stabilization and evacuation procedures, and casualty care at different levels of the medical evacuation chain. Participants were also trained to operate as combat medics at the squad, platoon, and company levels, with a focus on performing under the demanding conditions of war.

Participants were surveyed immediately before ORT classroom training (T1), immediately after ORT classroom training (T2), and a month later at the end of the five-week medic course (T3). The surveys were linked using self-generated codes rather than names or assigned identifiers to preserve anonymity in a sensitive wartime training context, and to avoid asking participants to remember assigned identification numbers across survey waves. Because the surveys were administered to course cohorts rather than individually tracked participants, and because self-generated codes were sometimes missing or inconsistent, only participants with matchable codes were included in longitudinal analyses. The T3 survey was administered only to a subset of the initial sample due to logistical constraints.

### Participants

The main study sample (*N* = 145; see Figure 1 for participant selection) consisted of Ukrainian service members who were enrolled in a five-week combat medic course in Norway and who completed surveys at both T1 and T2. Only consenting participants who had an anonymous code that could be linked between T1 and T2 were included for analysis (145/185; 78.4% of the T1 sample). A sub-group of participants (*n* = 39) were matched across T1, T2 and T3. We note that one individual withdrew from the study at T3.

**Figure 1.**
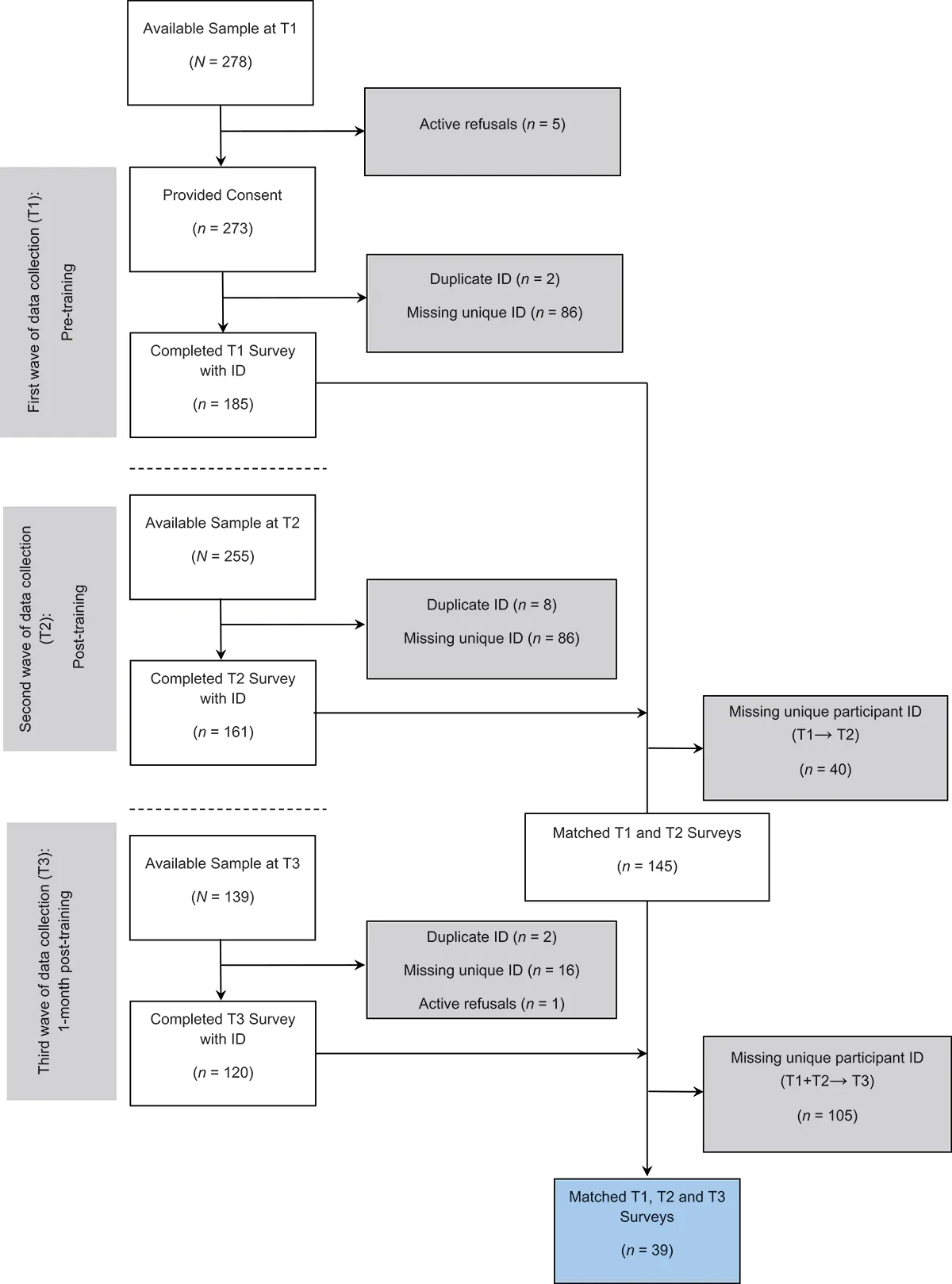
Participant flow chart.

### Training

ORT content had to be selected and adapted quickly because only weeks elapsed between the initial request for training and the arrival of the first Ukrainian service members for the combat medic course in Norway. Thus, the ORT development team (three of the authors: MASKED) selected established modules that had previously been developed, piloted, and evaluated with service members as the foundation for the program. Ukrainian military leaders and consultants then reviewed early versions of the material. Their feedback led the team to retain and tailor a module on managing stress during exposure to human remains and to add a module on combat-related grief, both of which addressed common and operationally salient frontline challenges. Although these specialized modules were grounded in scientific literature and operational experience, they required more original adaptation than the established modules.

Ultimately, seven domains were covered: (1) fundamentals of stress and resilience (Adler et al., 2026; Nordstrand, Engen, et al., 2024), (2) forward military mental health (Nugent et al., 2024), (3) operational stress control (Adler et al., 2015, 2021), (4) acute stress reactions (Adler & Gutierrez, 2022), and (5) cohesion and tactical communication (Cacioppo et al., 2015; Crouch et al., 2024) (6) frontline loss and grief, and (7) mental skills for working with human remains. Details regarding the training content are provided in Table 1, and the accompanying pocket guides are located at https://www.forsvaret.no/en/news/articles/new-manuals-in-operational-resilience-training in Norwegian, English, and Ukrainian.

**Table 1.** Operational Resilience Training modules and examples of content.

Module	Objective	Examples of content and adaptation
Fundamentals of stress and resilience	Normalize combat and deployment stress reactions; introduce the domains of psychological resilience, the stress bucket metaphor and the stress reactions continuum.	Ukrainian frontline examples, including drone threat, sustained uncertainty, casualty exposure, and harsh living conditions
Forward military mental health	Recognize common mental health concerns and provide first-line support without pathologizing normal stress responses.	Brief behavioral health literacy, stigma reduction, active listening, cognitive behavioral techniques to identify common thinking traps, meaning-making, moral injury mitigation, and referral considerations when formal resources are limited
Operational stress control	Strengthen mental preparation, self-regulation and performance under pressure, and ability for post-combat recovery.	Grounding, attention control, self-talk, tactical breathing, visualization, cue-words, and brief recovery routines after exposure such as optimizing sleep in a field setting, gratitude practices and progressive muscle relaxation
Acute stress reactions/iCOVER	Recognize and respond to acute stress reactions with the iCOVER peer-based intervention steps.	Training iCOVER using a craw-walk-run framework with theoretical instruction and progressively challenging field-based scenarios
Cohesion and tactical communication	Increase unit connection, shaping unit micro-culture, and effective communication	Practical cohesion-building behaviors, written and unwritten cultural rules, integration of new service members, clear communication in the field, closed loop communication, and rules of engagement for managing interpersonal disagreements
Frontline grief	Support peers grieving deaths, injuries, and ambiguous losses.	Understanding grief reactions and dispelling myths, distinguishing grief from depression, and providing support to grieving unit members by acknowledging loss, preserving a sense of connection to lost team members, and returning to a mission focus without dismissing grief
Mental skills for working with human remains	Maintain functioning and protect mental state during and after exposure to human remains.	Steps for mental preparation before exposure, activation regulation, attention control techniques, buddy care and sustainment practices during exposure, and managing sensory memory, meaning-making and social support after exposure.

Note. iCOVER = identify, connect, offer commitment, verify facts, establish order, and request action.

Two overarching concepts were consistently integrated into the training. First, stress was described as a metaphorical bucket that fills up and can overflow, overwhelming the individual, unless coping mechanisms drain the bucket (Nordstrand, Engen, et al., 2024). Second, stress reactions were conceptualized along a color-coded continuum that enables medics to evaluate soldiers’ stress levels from “optimal readiness” to “combat ineffective” and determine a range of possible associated interventions (Adler et al., 2026).

ORT materials included Ukrainian-specific references, images, videos, and points of emphasis, consistent with feedback from Ukrainian experts. Ukrainian experts included a psychiatrist serving in the Ukrainian Armed Forces who had supported operational units near the frontlines, a Ukrainian psychotherapist and veteran with frontline experience, and a Ukrainian mental health influencer with extensive combat experience as a soldier in the infantry. The cultural adaptation included images and examples reflecting Ukrainian field conditions, video material featuring Ukrainian service members, and case examples involving drone threat, casualty care, grief following unit losses, and exposure to human remains. The training also emphasized self-care and buddy-care strategies that medics could use in settings where formal mental health resources were limited or difficult to access.

The training was delivered in English by Norwegian mental health officers with advanced degrees in psychology, with simultaneous translation into Ukrainian and supported by Ukrainian-language slides and handouts. Each course was conducted by two to three ORT instructors (not the same personnel providing the medical components of the combat medic course). Training sessions occurred in groups of approximately 30 Ukrainian service members. All ORT trainers were instructed and supervised by either the ORT developers or experienced ORT trainers; detailed instructor notes accompanied each slide.

### Measures

Demographic questions (i.e., age category, highest level of educational attainment, months in the military, and combat experience) were included on the T1 survey. Attitudes toward training were assessed at T2 with five items (e.g., “The training was useful,” “I will most likely use the resilience skills”). Participants rated each item using response options ranging from 1 (strongly disagree) to 5 (strongly agree). Similar items have been used in prior research evaluating mental skills training programs for military personnel (Adler et al., 2020; Foran et al., 2012).

Confidence was assessed at T1, T2, and T3 with nine items using response options ranging from 1 (strongly disagree) to 5 (strongly agree). The items were developed for this study to reflect domains covered by the ORT training modules and were adapted from previous research assessing confidence in mental skills in military samples (Adler & Gutierrez, 2022). Four items reflected confidence regarding an individual’s own skills (e.g., “I am confident I can improve my own combat effectiveness”) and five items reflected confidence in supporting others (e.g., “I am confident I can help others with combat stress”).

### Statistical analyses

Frequencies and percentages were reported for demographics and training acceptability items. To examine change over time, the percentage of participants reporting high confidence before ORT classroom training (T1) and immediately after training (T2) was calculated.

Confidence responses were dichotomized into non-endorsement (scores 1–3) and endorsement (scores 4–5) to facilitate interpretation because the study aimed to examine whether participants endorsed confidence rather than the degree of confidence. Only clear agreement (“agree” or “strongly agree”) was counted as endorsement, and the neutral midpoint (3) was included in the non-endorsement category (strongly disagree and disagree) to ensure a conservative estimate.

McNemar’s test was used to assess differences in paired categorical responses from T1 to T2. Odds ratios (ORs) and 95% confidence intervals (CIs) were calculated to estimate the strength of the association between ORT training and change in confidence classification. Next, an exploratory sub-analysis was performed with the T1-T3 cohort to assess changes from T2 (post-training) to T3 (end of the five-week medic course) using McNemar’s test. This analysis explored whether there were differences in paired categorical responses from T2 to T3. All statistical analyses were conducted using Stata version 18 (StataCorp, 2023), with statistical significance set at *p* values ≤ .05 (two-sided).

## Results

Of the 145 participants matched between T1 and T2, most were under 40 years old (40.0% were less than 30 years old, and 37.9% were 30–39 years old) and had served either 0–12 months (36.9%) or 12–24 months (36.2%) in the military. The majority of the sample (81.3%) had finished at least secondary education. Descriptive and military characteristics of the service members are summarized in Table 2.

**Table 2.** Demographic characteristics of Ukrainian service members in the sample.

	No. (%)
Demographic variables	Study sample (*n* = 145)
Age	
<30	58 (40.0%)
30–39	55 (37.9%)
40–49	27 (18.6%)
50–59	5 (3.4%)
Educational attainment	
Basic secondary education (9th grade)	5 (3.5%)
Specialized secondary education (11th grade)	22 (15.2%)
Some university or technical coursework	6 (4.0%)
Technical or vocational school	71 (49.0%)
Bachelor’s degree	23 (15.9%)
Master’s degree or more	18 (12.4%)
Months in the Military	
0–12 months	52 (36.9%)
12–24 months	51 (36.2%)
24–48 months	22 (15.6%)
48+ months	16 (11.3%)
Combat experience	
No	43 (29.7%)
Yes	101 (69.7%)

Note. Some columns do not add up to total sample size due to missing data.

Most participants rated ORT as acceptable, as shown in Table 3. Nearly all participants found the training useful (98.6%), relevant (96.6%), and important (93.8%). Additionally, 94.5% indicated they would likely use the resilience skills learned in the future.

**Table 3.** Attitudes toward Operational Resilience Training among Ukrainian service members.

	% (*n*)
Attitude items	Study sample
This training was useful	98.6% (143)
This training was relevant	96.6% (140)
This training was important	93.8% (135)
I will most likely use the resilience skills I have learned in the future	94.5% (137)

Note. The numbers represent participants who either agree or strongly agree with each item. Some columns may not sum to the total study population due to missing data.

Table 4 presents the results of McNemar’s test related to changes in the proportion of participants endorsing confidence in applying mental skills, with comparisons between pre-training (T1) and immediate post-training (T2). The results revealed significant increases in the proportion of participants endorsing confidence in using ORT mental skills to help themselves. Specifically, there were significant increases from pretest to posttest in the ability to improve their own combat effectiveness (an increase of 9.1% points), χ^2^ (1) = 6.26, *p* = .01; tactical communication skills (an increase of 30.0% points), χ^2^ (1) = 39.34, *p* < .001; helping themselves with combat stress (an increase of 16.8% points), χ^2^ (1) = 14.40, *p* < .001; and protecting their mental state when dealing with human remains (an increase of 23.7% points), χ^2^ (1) = 24.08, *p* < .001. Figure 2 summarizes these percentage-point changes.

**Figure 2.**
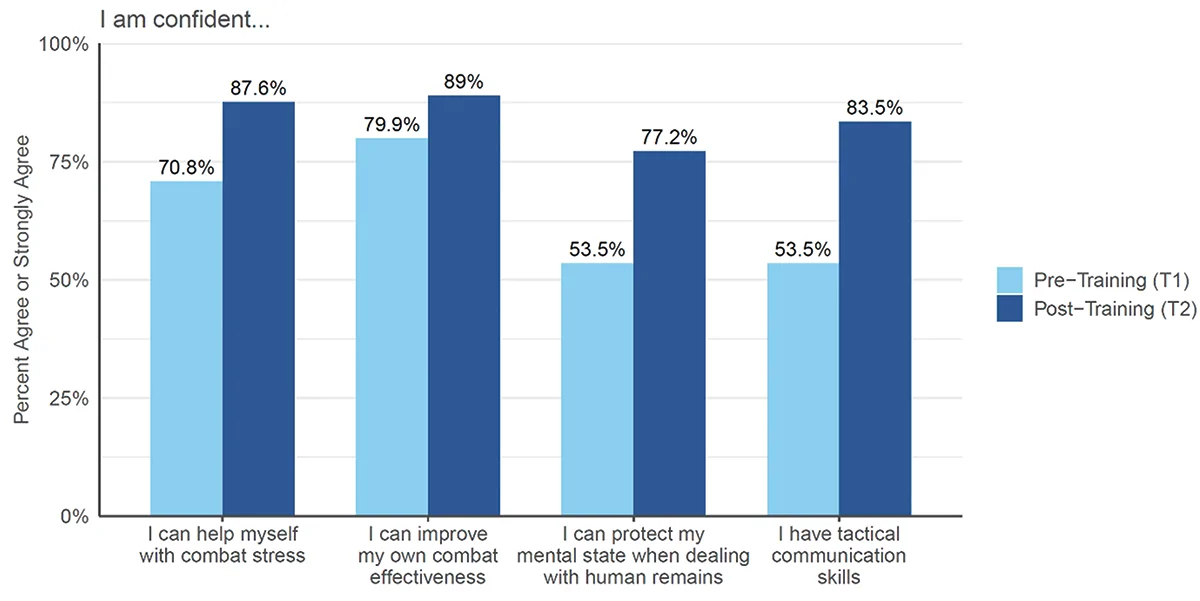
Participant ratings of confidence in skills for helping themselves before and after Operational Resilience Training.

**Table 4.** Odds ratios for changes in confidence skills among Ukrainian service members (pre-training vs. post-training).

Confidence items	OR (95% CI)	*p*-value
Mental skills for personal use		
I am confident I can improve my own combat effectiveness	2.86 [1.16, 8.00]	.01
I am confident I have tactical communication skills	22.5 [5.88, 191.47]	<.001
I am confident I can help myself with combat stress	4.00 [1.81, 10.05]	<.001
I am confident I can protect my mental state when dealing with human remains	5.86 [2.60, 15.47]	<.001
Mental skills for using with others		
I am confident I can improve others’ combat effectiveness	7.00 [2.73, 22.89]	<.001
I am confident I have skills to increase unit cohesion	11.40 [4.61, 36.45]	<.001
I am confident I can help others with combat stress	8.40 [3.33, 27.29]	<.001
I am confident I can help others who are having an ASR	14.33 [4.59, 72.22]	<.001
I am confident I can help others who are grieving	2.62 [1.35, 5.40]	.002

There were also significant increases from pretest to posttest in the proportion of participants endorsing confidence related to using mental skills to help others. These increases included improving the combat effectiveness of others (an increase of 21.0% points), χ^2^ (1) = 22.50, *p* < .001; increasing unit cohesion (an increase of 36.3% points), χ^2^ (1) = 43.61, *p* < .001; helping others with combat stress (an increase of 26.9% points), χ^2^ (1) = 29.13, *p* < .001; helping others who are having an acute stress reaction (ASR; an increase of 28.3% points), χ^2^ (1) = 34.78, *p* < .001; and helping others who are grieving (an increase of 14.4% points), χ^2^ (1) = 9.38, *p* = .002. Figure 3 summarizes these percentage-point changes.

**Figure 3.**
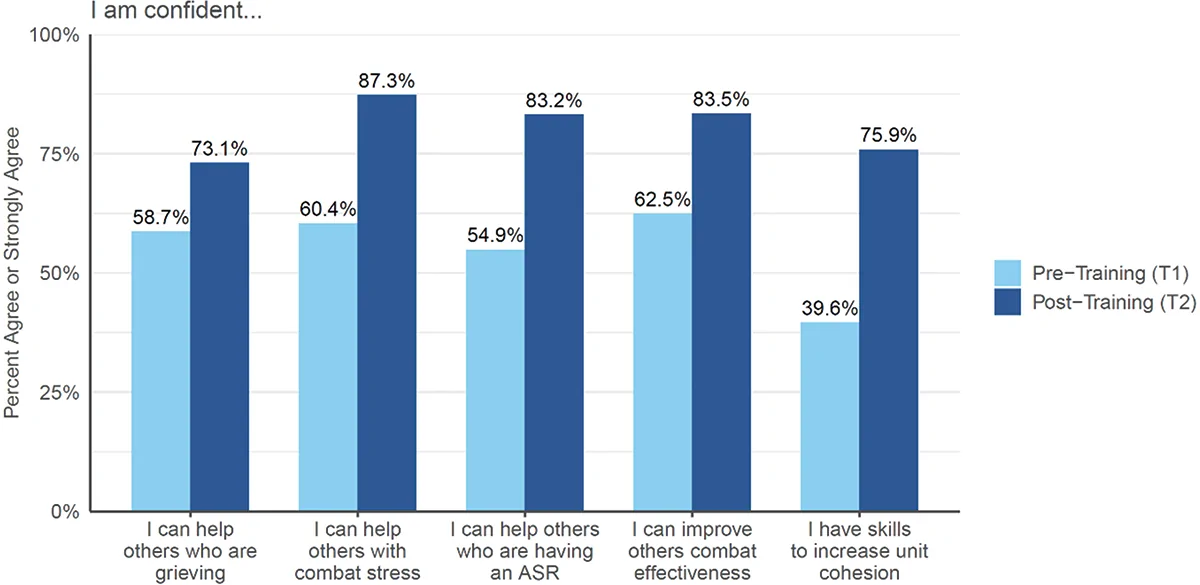
Participant ratings of confidence in skills for helping others before and after Operational Resilience Training.

An analysis of the subsample with survey results matched between T2 and T3 (*n* = 39) found no significant changes in any of the confidence items, indicating that participants’ confidence levels for helping themselves and others remained stable over this period.

## Discussion

In the current study of Ukrainian service members who completed a combat medic course, resilience training aimed at enhancing their capacity to withstand psychological stress during frontline deployment and combat received positive ratings and was associated with increases in confidence over the combat medic course training period. Nearly all surveyed service members found the training useful, relevant, and important, and expressed intentions to apply the skills they acquired. Furthermore, participants reported greater confidence in managing personal stress and supporting others. For instance, there was an increase of 30.0% points in endorsement of confidence regarding tactical communication skills and an increase of 36.3% points in endorsement of confidence regarding skills aimed at enhancing unit cohesion.

The positive feedback on the ORT course, combined with the finding that nearly 70% of participants had frontline combat experience, suggests that resilience and mental skills training may provide relevant support even to seasoned combat veterans. At the same time, the results should not be interpreted as evidence that ORT necessarily improved competence in resilience skills. The measures assessed confidence in using ORT-relevant skills, not observed skill performance or implementation under operational conditions. With that qualification, the study suggests that ORT may offer a scalable training model for increasing service members’ confidence in managing various critical domains. These domains range from acute stress management, effective communication, and performance under pressure, to grief management and working with human remains. Thus, even as part of a rapid mobilization typical of large-scale combat operations, resilience training may be able to contribute to service member confidence in ways that could contribute to broader military readiness.

A further contribution of the present study is the documentation of a rapidly adapted, modular approach to mental skills training. Most ORT modules drew on established training programs, whereas the human remains and grief domains required more original tailoring to Ukrainian operational needs. Although analyses related to these new modules should therefore be interpreted cautiously, they were also associated with increased confidence. Specifically, participants showed an increase of 23.7% points in confidence that they could protect their mental state when working with human remains and an increase of 14.4% points in confidence that they could help others who were grieving. These findings suggest the feasibility of developing targeted modules to address rapidly evolving psychological demands, although more rigorous evaluation of these modules is needed.

### Limitations and future directions

While the current study offers insights into the experiences and needs of service members operating under extreme occupational conditions involving direct and severe threats, several limitations should be acknowledged. First, because the evaluation did not employ a randomized design, alternative factors may have contributed to the observed increase in participants’ confidence. Future efforts should use randomized controlled designs to evaluate ORT when operationally feasible.

Second, there was substantial study attrition. In all, 21.6% of T1 participants could not be matched to T2 when surveyed the following day. This loss of data was likely influenced by the decision to protect anonymity through self-generated codes, the reality that surveys were administered by course cohort rather than through individually tracked appointments, and fact that there were competing demands for participant time during the tightly scheduled medic course. The attrition rates introduce the potential for bias in participant retention and should be addressed in future studies through improved linking procedures and research conditions that promote higher participant retention. The follow-up sample at T3 was also small because logistical constraints in the field-training context resulted in T3 data for only a subset of respondents, and the long follow-up period might have led participants to forget which individualized code they had selected at T1 and T2. This reduced the statistical power of the follow-up analyses, and thus these results are only regarded as preliminary.

Third, all measures relied on self-report, and no objective assessments were included to evaluate the actual implementation of ORT techniques and skills. Future research should leverage performance-based outcomes, instructor ratings, peer ratings, or behavioral indicators of skill use.

Fourth, due to practical constraints requiring a brief survey, it was not feasible to include detailed items about the specific combat history of participants. Future research should examine the relationship between training response and individual differences in combat experience, deployment history, conscript versus volunteer status, and occupational specialty.

Fifth, dichotomizing the Likert-scale responses reduced variability and potentially resulted in a loss of information, although the more conservative approach of distinguishing between those who were confident from those who were not allowed for a clearer contrast in changes over time. Finally, given the real-world nature of the evaluation, follow-up surveys after participants returned to their military units in Ukraine were not feasible, limiting insight into how effectively ORT training was integrated into their military duties.

Despite these limitations, evidence from the field suggests ORT is seen as valuable. The National Guard of Ukraine has established a mandatory 1-day ORT course for all service members (Nordstrand et al., 2026), focusing on individual (rather than medic-oriented) skills. This course implementation and dedication of resources and training time supports the interpretation that the Ukrainian Armed Forces regard ORT as a useful part of supporting readiness in a large-scale combat operation.

Given that the present modules were selected from a larger set of possible mental skills under urgent operational conditions, future research should compare alternative module combinations, identify which skills are essential versus optional, and test whether different operational roles require different configurations. ORT could also be adapted for other occupational roles beyond combat medics, including leaders, mortuary affairs personnel, and special forces. Future research could also consider building on this study’s initial findings by expanding training in addressing death anxiety, moral awareness (Gutierrez et al., 2024), and suicide prevention (Skrindo & Sæheim, 2019).

Further work should examine whether ORT-trained combat medics are sufficient as first-line psychological support providers or whether the addition of behavioral health medics or other frontline mental health personnel improves outcomes. Moreover, developing systematic guidelines for cultural adaptation may enhance the effectiveness of ORT across diverse contexts. Studies could also evaluate whether ORT materials remain applicable and beneficial in peacekeeping operations and humanitarian missions.

## Conclusions

The present evaluation indicates that resilience training can be perceived as relevant and useful by service members facing extreme circumstances. An important feature of this training was the modular structure, allowing adaptation and updates to fit specific cultural and operational contexts. Additionally, by transitioning from classroom instruction to practical field exercises, the training demonstrated how mental skills can be developed similarly to other performance-based tasks commonly trained in military contexts (Nordstrand et al., 2026; Thompson & Jetly, 2014; Tornero-Aguilera et al., 2024). These insights can inform improvements in resilience training within the Ukrainian Armed Forces as well as other military organizations. By emphasizing practical application, integrating resilience skills with occupational demands, and applying evidence-based recommendations, Operational Resilience Training may help combat medics and those they support manage stress in extreme environments.

## Supplementary Material

Public Significance Statement.docx

## Data Availability

Data can be accessed by contacting the authors directly.
