# Supplementary material for: Psychological resilience on the frontlines: Operational Resilience Training for Ukrainian combat medics
Source: Mil Psychol. 2026 Jul 2;38(6):926–36. doi: 10.1080/08995605.2026.2698195 (PMC13750245; doi:10.1080/08995605.2026.2698195)
Supplement: Supplementary file 1 [file HMLP_A_2698195_SM3895.docx]

What is the public significance of this article?

Combat medics in large-scale combat operations may need practical tools to support both physical survival and psychological functioning at the frontlines. This study suggests that Operational Resilience Training was viewed as useful and relevant by Ukrainian combat medics and was associated with increased confidence in using mental skills for self-care and peer support. These findings may help military organizations develop scalable resilience training for medics and other frontline personnel, while underscoring the need for future studies using performance-based outcomes.
